# 

**Published:** 1879-09

**Authors:** 


MEDICAL COLLEGES. — Before another number of our journal will reach its readers, the annual lecture terms in the medical schools in this city will have commenced. The general introductory lecture to the course in the Chicago Medical College will be on the evening of September 30; that in the Rush Medical College on the evening of September 30; and the first lecture of the course in the Woman's Medical College will be at 10 a.m., of the first Tuesday in October.

### BOOKS AND PAMPHLETS RECEIVED.

Manual of the Principles and Practice of Operative Surgery. By Stephen Smith, A.M., M.D. Cl., pp. 689; 1879. Boston: Houghton, Osgood & Co.

A Clinical Treatise on the Diseases of the Nervous System. By M. Rosenthal; with preface by Professor Charcot. Translated from the author's revised and enlarged edition by L. Putzel, M.D. Cl., pp. 278; 1879. New York: Wm. Wood & Co. Chicago: W. T. Keener.

Transactions of the Medical Society of the State of Tennessee, at its Forty-sixth Annual Meeting, 1879.

Posture as a Means of Relief in Strangulated and Incarcerated Hernia, with a General Consideration of the Mechanism of Reduction. By F. H. Hamilton, A.M., M.D. Reprint from *Hospital Gazette*, June 7, 1879.

An Account of the Perineosinnexereenatur—A New Instrument for the Exploration of Sinuses, especially adapted to Gynæcological Practice. By Jacques Robinson, A.M., M.D. Reprint from *Louisville Med. News*, May 13, 1879.

Observations on Amphoric Respiration and Amphoric Respiratory Echo. By M. L. James, M.D.

The Treatment of Epithelioma of the Cervix Uteri. By J. Marion Sims, M.D. Reprint from *Amer. Jour. of Obstetrics and Diseases of Children*, July, 1879.

Twelfth Biennial Report of the Trustees, Superintendent and Treasurer of the Tennessee Hospital for Insane, Jan. 8, 1879.

Medical Heroism of 1878. By J. W. Singleton, M.D. Reprint from *St. Louis Medical and Surgical Journal*, June, 1879.

Chronic Spasmodic Stricture or Urethrismus. Second paper in reply to Dr. H. B. Sands. By F. N. Otis, M.D. Reprint from *Hospital Gazette*, June 28, 1879.

Early Medical Chicago. By J. N. Hyde, A.M., M.D.

Minutes of Medical Society of the County of New York, 1806–1878. Edited by A. E. M. Purdy, M.D. July; Part IV.

Retroversion in Relation to Laceration of the Cervix Uteri, etc. By Nathan Bozeman, M.D. Reprint from Vol. III, Gynæcological Trans., 1879.

History of the Discovery of Anæsthesia. By J. M. Sims, M.D., LL.D. From *Virg. Med. Monthly*, May, 1879.

Reflex Cerebral Hyperæmia. By C. H. Hughes, M.D. Reprint from *St. Louis Med. and Surg. Jour.*

Pure Rubber Martin Bandages. Reprint from *Chic. Med. Jour. and Ex.*, Aug., 1879.

---

YELLOW FEVER. — This disease continues its steady progress in Memphis, and develops here and there a case in smaller country places, just as though it was deliberately showing its contempt for all the quarantines, disinfectants, fumigations and isolations, that the advocate of *germ theories* can invent. So too, in New Orleans, cases of the disease occur sporadically once in a few days just as has always been the case in seasons when the epidemic influence was not strong and in the same parts of the city. How much longer will it take the profession to learn that the best disinfectant is pure air, and the best antidote for epidemics, pure air, pure water, cleanliness of person, premises and soil?

KENTUCKY-LOUISVILLE MEDICAL COLLEGES. — These two schools, for many years claiming to be distinct and under separate charters, but run by one faculty and in one building, have finally discontinued their suspicious relationship, and hereafter each will have its own faculty and building.

### ANNOUNCEMENTS FOR THE MONTH.

### SOCIETY MEETINGS.

Chicago Medical Society—Mondays, Sept. 1 and 15.

West Chicago Medical Society—Mondays, Sept. 8 and 22.

### CLINICS.

MONDAY.

Eye and Ear Infirmary—2 p. m., Ophthalmological, by Prof. Holmes; 3 p. m., Otological, by Prof. Jones.

Mercy Hospital—1:30 p. m., Surgical, by Prof. Andrews.

Rush Medical College—2 p. m., Dermatological and Venereal, by Prof. Hyde; 3 p. m., Medical, by Dr. Bridge.

Woman's Medical College—2 p. m., Dermatological, by Dr. Maynard.

TUESDAY.

Cook County Hospital—2 to 4 p. m., Medical and Surgical Clinics.

Mercy Hospital—1:30 p. m., Medical, by Prof. Hollister.

WEDNESDAY.

Chicago Medical College—1:30 p. m., Eye and Ear, by Prof. Jones.

Rush Medical College—3:30 to 4:30 p. m., Diseases of the Chest, by Dr. E. Fletcher Ingals.

THURSDAY.

Chicago Medical College—1:30 p. m., Medical, by Prof. Quine.

Rush Medical College—3 p. m., Diseases of the Nervous System, by Prof. Lyman.

Eye and Ear Infirmary—2 p. m., Ophthalmological, by Dr. Hotz.

FRIDAY.

Cook County Hospital—2 to 4 p. m., Medical and Surgical Clinics.

Mercy Hospital—1:30 p. m., Medical, by Prof. Davis.

SATURDAY.

Rush Medical College—2 p. m., Surgical, by Prof. Gunn.

Chicago Medical College—2 p. m., Surgical, by Prof. Isham; 3 p. m., Neurological, by Prof. Jewell.

Woman's Medical College—11 a. m., Ophthalmological by, Dr. Montgomery.

Daily Clinics, from 2 to 4 p. m., at the Central Free Dispensary, and at the South Side Dispensary.

